# The role of purinergic receptors in stem cell differentiation

**DOI:** 10.1016/j.csbj.2014.11.003

**Published:** 2014-11-07

**Authors:** Constanze Kaebisch, Dorothee Schipper, Patrick Babczyk, Edda Tobiasch

**Affiliations:** Department of Natural Sciences, Bonn-Rhine-Sieg University of Applied Sciences, Von-Liebig-Str. 20, 53359 Rheinbach, Germany

**Keywords:** P1 receptor, P2 receptor, Purinergic signaling, Regenerative medicine, Adipose tissue-derived stem cells, Bone marrow-derived stem cells

## Abstract

A major challenge modern society has to face is the increasing need for tissue regeneration due to degenerative diseases or tumors, but also accidents or warlike conflicts. There is great hope that stem cell-based therapies might improve current treatments of cardiovascular diseases, osteochondral defects or nerve injury due to the unique properties of stem cells such as their self-renewal and differentiation potential. Since embryonic stem cells raise severe ethical concerns and are prone to teratoma formation, adult stem cells are still in the focus of research. Emphasis is placed on cellular signaling within these cells and in between them for a better understanding of the complex processes regulating stem cell fate. One of the oldest signaling systems is based on nucleotides as ligands for purinergic receptors playing an important role in a huge variety of cellular processes such as proliferation, migration and differentiation. Besides their natural ligands, several artificial agonists and antagonists have been identified for P1 and P2 receptors and are already used as drugs. This review outlines purinergic receptor expression and signaling in stem cells metabolism. We will briefly describe current findings in embryonic and induced pluripotent stem cells as well as in cancer-, hematopoietic-, and neural crest-derived stem cells. The major focus will be placed on recent findings of purinergic signaling in mesenchymal stem cells addressed in *in vitro* and *in vivo* studies, since stem cell fate might be manipulated by this system guiding differentiation towards the desired lineage in the future.

## 1 Introduction

Over the last decades, stem cells have received considerable attention due to their ability of self-renewal and their capacity to differentiate into a wide range of specialized cell types [1]. They have been extensively studied with respect to their applicability for treating a variety of clinical pathologies such as myocardial infarction [2] or critical size bone defects [3]. Especially in the field of tissue reconstruction and transplantation, stem cell-based approaches display a promising tool for which mesenchymal stem cells (MSCs) represent an attractive cell source. Although there are numerous publications illustrating the interaction of extracellular nucleotides and purinergic receptors, little is known about their particular role in embryonic or adult stem cells. Upon binding to their natural ligands purinergic receptors implement a variety of biological actions in many cell and tissue types [4]. Even though it is known that purinergic downstream signaling plays an important role in cellular processes such as proliferation, migration, and differentiation, more detailed insights into these processes are obligatory for the establishment of future clinical applications using the differentiation potential of stem cells without undesired side effects [5]. This review will outline the current state of knowledge on the role of purinergic receptors and their ligands in different pluripotent and multipotent stem cell types with main focus on MSC proliferation and differentiation.

## 2 Purinergic receptors—structure and distribution

**Fig. 1:**
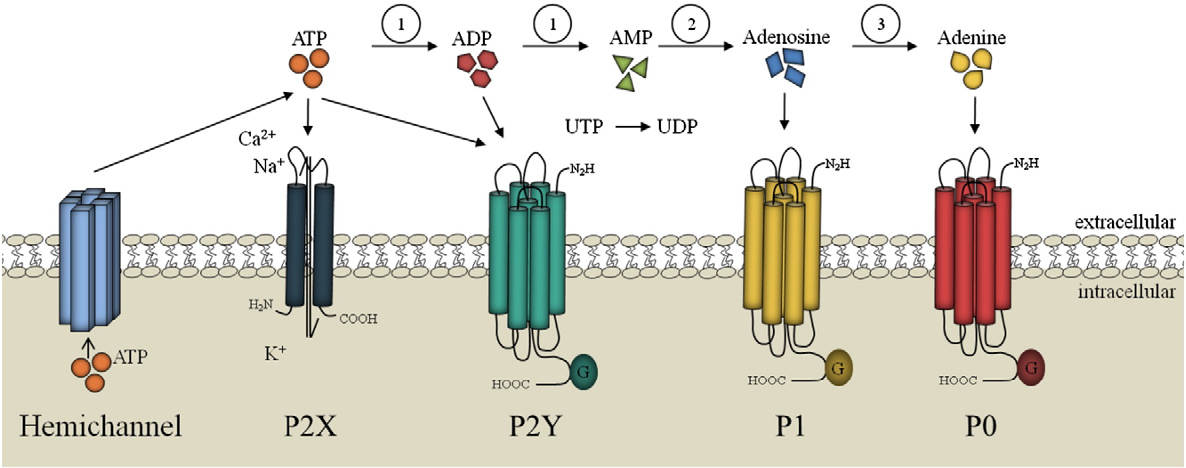
Purinergic receptors and their natural ligands. Purinergic receptors are divided into P2 receptors which are activated by a variety of nucleotides and can be further subdivided into ionotropic P2X receptors activated by ATP and the metabotropic G-protein-coupled receptors (P2Y) which are stimulated by nucleotides, di- or triphosphates, purines or pyrimidines. In contrast, metabotropic P1 receptors are preferentially activated by adenosine. Recently, evidences for the functional expression of adenine receptors, designated as P0 receptors, have been found. 1: ecto-nucleoside triphosphate diphosphohydrolases (E-NTPDases) e.g. CD39, 2: ecto-5’-nucleotidase (CD73), 3: purine nucleoside phosphorylase (PNP).

Purinergic receptors are one of the evolutionary oldest receptors [9]. The receptor family can be found in almost every mammalian tissue and was initially described in gut smooth muscle cells in the 1970s[7], [8]. In 2014 the first purinergic receptor, namely DORN1, was discovered in plants [6]. Purinergic receptors are divided into P1 receptors which are preferentially activated by adenosine and P2 receptors which are activated by a variety of nucleotides. The latter ones are subdivided into ligand-gated ion channels (P2X) activated by ATP and G-protein-coupled receptors (P2Y) which are activated by nucleotides, di- or triphosphates, purines or pyrimidines (see Fig. 1) [10]. ATP released from cells by several mechanisms e.g. mechanical stimulation is rapidly degraded to adenosine by ectonucleotidases [4]. This ligand receptor system takes part in neurotransmission, mechanosensory transduction, secretion and vasodilatation, as well as long-term signaling functions in cell proliferation, differentiation, and death [4]. Recently, evidence for the functional expression of adenine receptors, designated as P0 receptors, has been found (Fig. 1)[11], [12].

### 2.1 P1 purinoreceptors

P1 receptors are G-protein-coupled receptors expressed in nearly all cell types and take part in a lot of physiological processes within the heart, the cardiovascular system, the nervous system, during inflammation and in pain. Adenosine acts as a natural ligand of P1 receptors and contributes to physiological processes such as cell proliferation and migration in endothelial cells [126]. The P1 receptors are structured into four receptor subtypes named A1, A2A, A2B and A3 and consist of seven transmembrane domains. Next to adenosine they can also be activated and inactivated by various artificial agonists and antagonists (for details see the review of Fredholm et al. [13]). P1 receptor expression in stem cells was reported by different working groups and the understanding of the contribution of these receptors to proliferation and differentiation is increasing [14], [15], [16].

### 2.2 P2 purinoreceptors

#### 2.2.1 P2X receptors

P2X receptors are cation-permeable ligand-gated ion channels which are activated by ATP. Almost every tissue and cell type shows regulated release of ATP mainly via vesicular or conductive mechanisms, whereby the latter ones involve nucleotide transport via hemichannels e.g. pannexins [131]. To date, seven receptor subtypes (P2X1-7) are known that form homomeric (P2X1-5) and heteromeric (P2X2/3 and P2X1/5) receptors, with the exception of P2X6 that cannot form functional homomeric and P2X7 that cannot form functional heteromeric receptors [17], [18]. Each subunit consists of two transmembrane domains separated by an approximately 280 amino acid extracellular domain. P2X receptors are abundantly distributed, and functional responses have been described for neurons, glia cells, epithelial cells, endothelial cells, bone, muscle, and hematopoietic tissues [19], [20]. The receptors are involved in a variety of physiological processes, e.g. the modulation of vascular tone, chronic pain, contraction of urinary bladder, platelet aggregation, macrophage activation, apoptosis and neuronal–glial interaction [21], [22], [23], [24]. The role of P2X receptors during proliferation of hMSCs was described by Coppi and colleagues and the involvement of P2X5 and P2X7 in osteogenic differentiation was shown by Zippel and coworkers [16], [25].

#### 2.2.2 P2Y receptors

P2Y receptors are G-protein-coupled receptors. In contrast to P1 receptors these receptors are activated by nucleotides like ATP, ADP, UTP, UDP and UDP-glucose [10]. There are eight subtypes known in human tissues named P2Y1, P2Y2, P2Y4, P2Y6, P2Y11, P2Y12, P2Y13, and P2Y14. The missing numbers represent either non-mammalian orthologs or receptors having some sequence homology without showing functional responses to nucleotides [26]. Investigations of P2Y1 and P2Y2 have shown that some positively charged residues in transmembrane domains (TM) 3, 6, and 7 are crucial for receptor activation by nucleotides and that they share a H-X-X-R/K motif in TM6 [27], [28]. The effect of P2Y receptor activation depends on the coupling to downstream signaling pathways, either via G_i_, G_q/11_ or G_s_ proteins. They are in the focus of many studies using agonists and antagonists for future drug development. For example they contribute to platelet aggregation (P2Y1 and P2Y12), pulmonary diseases (P2Y2 and P2Y4), hematopoiesis and immunity (P2Y11) [10], [26]. P2Y receptors have been reported to be involved in the adipogenic and osteogenic differentiation of hMSCs as well as the differentiation towards vascular lineages [25], [29].

## 3 Stem cells

**Fig. 2:**
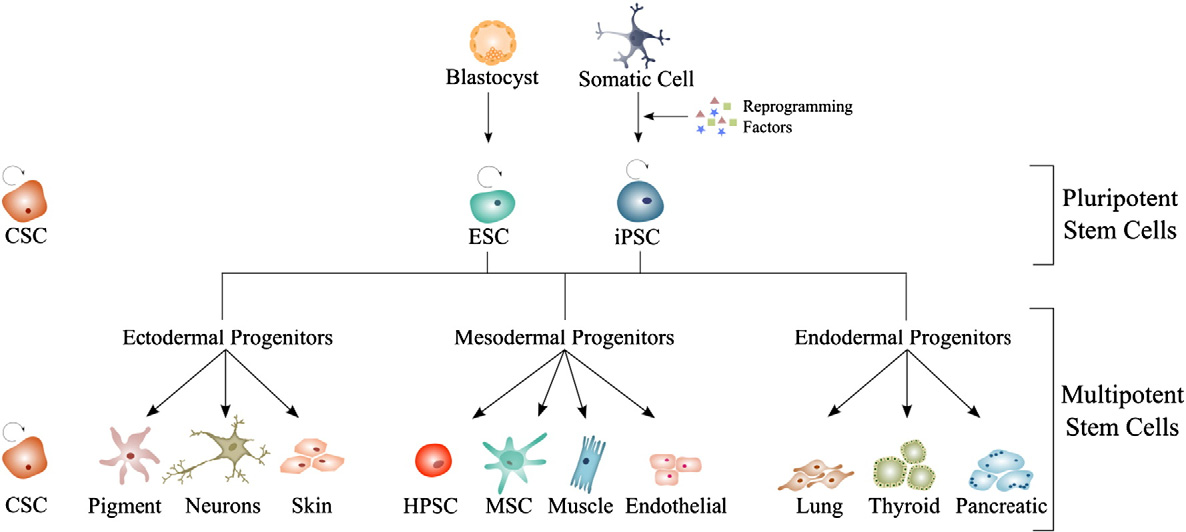
Differentiation potential of pluripotent stem cells. Pluripotent stem cells like embryonic stem cells (ESC), cancer stem cells (CSCs) or induced pluripotent stem cells (iPSC) are able to differentiate into all cell types of the three germ layers: ectoderm (such as pigment, neuronal and skin cells), mesoderm (such as hematopoietic and mesenchymal stem cells or muscle and endothelial progenitor cells) and endoderm (such as lung-, thyroid- and pancreatic cells).

Stem cells are pluripotent or multipotent cells with two typical features: the capability of self-renewal and the potential to differentiate towards cell types of the human body. Depending on the source they are derived from, stem cells can be classified into embryonic and adult stem cells, the latter comprising mainly hematopoietic, neural crest-derived and mesenchymal stem cells. Recently, a new source of stem cells has been created, known as induced pluripotent stem cells. Another controversial discussed issue is the existence of so called cancer stem cells (see Fig. 2).

### 3.1 Pluripotent stem cells

#### 3.1.1 Embryonic stem cells

Embryonic stem cells (ESCs) are derived from the inner cell mass of blastocysts. The advantage of pluripotency and the potential to differentiate into cells of all three germ layers is coupled with several disadvantages, such as ethical problems and teratoma formation [30], [31]. Purinergic signaling in ESCs has been described by Burnstock and Ulrich [32] in 2011. In mouse embryonic stem cells P2 receptors seem to be crucial for proliferation [33], [34].

#### 3.1.2 Induced pluripotent stem cells

Takahashi and colleagues [35], [36] induced pluripotency in mouse embryonic and adult fibroblasts via viral introduction of the transcription factors Oct3/4, Sox2, c-Myc and Klf-4. The obtained cells showed ESC-like morphology. Wernig and coworkers [37] showed that induced pluripotent stem (iPS) cells are similar but not identical to ESCs regarding methylation and the chromatin state. Stadtfeld's group [38] used nonintegrating adenoviruses transiently expressing the stem cell factors Oct3/4, Sox2, Klf4, and c-Myc. Kim and coworkers [39] only used the factors Oct3/4, and c-Myc or Klf4. Yu and colleagues [40] induced pluripotency and an ESC phenotype in human somatic cells and differentiated the cells into cells of the three germ layers. In the meantime, iPS cells induced with two factors (Oct3/4 and Sox2) and even with only one factor (Oct3/4) have been produced. The number of necessary factors depends on the cells source. Progenitor cells or even stem cells need fewer transcription factors for the reprogramming process [41]. First evidence for purinergic signaling in human iPS cells was reported by the group of Mastrangelo [42] in 2012.

### 3.2 Cancer stem cells

Despite immense progress in the clinical treatment of various types of cancer, resistance to chemotherapeutic drugs, cancer metastasis and tumor recurrence are important issues in oncology. A recent hypothesis suggests that these difficulties are related to cancer stem cells (CSCs) within the tumor [43]. This small population of cells is thought to be responsible for tumor propagation and maintenance due to its self-renewal capacity and multilineage differentiation potential [44], [45]. In contrast to normal tissue renewal, the cancer precursor cells fail to undergo maturation and thus accumulate, resulting in tumor formation [46]. This new paradigm of cellular differentiation is now understood as a plastic phenotypic shift of cancer cells into more primitive cells with stem-like properties [47].

CSCs can be identified based on the expression of specific cell surface markers such as CD24, CD29, CD44, CD133, and CD166 [48]. Particularly with regard to cancer treatment strategies targeting CSC-associated marker proteins, it has to be considered that the expression level and nature of these markers is very heterogeneous [49].

A CSC subpopulation that is substantial for survival of an aggressive tumor in the CNS can be obtained from glioblastoma [50]. Morrone and colleagues [51] studied several models of glioma tumor growth and suggested that endogenous ATP release can induce glioma cell proliferation via both, P1 and P2 purinoreceptor signaling. In addition, ATP-mediated signaling was shown to be essential during neuronal differentiation of the murine embryonal carcinoma cell line P19 [52]. Based upon these findings, Ledur and coworkers [50] investigated the role of ATP and purinergic receptors in human and rat glioma CSCs. They could show that ATP treatment altered the expression pattern of purinergic receptors compared to adherent cells and decreased tumor sphere formation. The P2X6 and P2X7 receptor subtypes were up-regulated in attached cells, whereas P2X4, P2Y1, and P2Y14 were found increased in tumor spheres. Further, ATP reduced the expression of the glioma CSC markers CD133, Oct-4, and Nanog indicating a decreased cancer stem cell population. Taken together, the purinergic system has to be considered as potential pharmacological target for cancer therapy [50], [53].

### 3.3 Multipotent stem cells

#### 3.3.1 Hematopoietic stem cells

Hematopoietic stem cells (HSCs) are multipotent adult stem cells that can differentiate into all types of mature blood cells such as macrophages, monocytes, dendritic cells, erythrocytes, lymphocytes, and platelets [54], [55]. They can be obtained from bone marrow, peripheral blood and umbilical cord/placenta blood and are characterized with positive expression of CD34, Thy1, CD133, and C-kit and lack of cell surface markers CD38, Lin, and CD45 [54], [56], [57]. With regard to adult stem cells, HSCs are among the best characterized and they are the only stem cells routinely used in the clinical setting [54].

So far, numerous data exist with regard to P1 and P2 receptor expression and signaling pathways in HSCs. Hofer and colleagues reported that adenosine A1 receptor stimulation inhibited whereas adenosine A3 receptor activation enhanced the proliferation of committed hematopoietic progenitor cells but showed no effect on more primitive cell populations indicating that adenosine receptor signaling is restricted to more mature cell compartments [58].

Next to P1 receptors, several functional P2 receptor subtypes have been found to be expressed in CD34 + HSCs [59]. Further, endogenous ATP release reduced hematopoietic progenitor proliferation and was therefore suggested to be a key regulator of HSCs pool size [60]. P2X receptors also seem to be involved in the differentiation of HSCs as their expression is up-regulated in early hematopoietic precursors from umbilical cord blood compared to adult human blood cells [61]. Based on data obtained from knockout mice studies, Cho and coworkers suggested that the P2Y14 receptor might be a key mediator for hematopoietic stem and progenitor cell regenerative response to tissue stress. Animals lacking this receptor subtype displayed enhanced hematological stress-induced cell senescence coincided with increased ROS, elevated p16(INK4a) expression, and hypophosphorylated Rb [132]. In the future, the animal model system might be utilized to achieve a better understanding for which regulatory molecules are involved in the onset of stress-induced senescence within the HSC compartment [133].

#### 3.3.2 Neural crest-derived stem cells

Neural crest-derived stem and progenitor cells have been identified not only in the central but also in the peripheral nervous system of vertebrates [62]. During embryogenesis multipotent neural crest cells invade almost all tissues, both neural and non-neural. Later they undergo differentiation towards neurons and glial cells of the peripheral nervous system as well as cells of the craniofacial skeleton, endocrine cells, and melanocytes or persist in adult organs and tissues [62], [63], [64]. Recently, neural crest derivates have also been found in adult bone marrow, carotid body and heart [65], [66], [67]. Stem cells in neural crest-derived adult tissues are characterized by the expression of diverse biomarkers such as the transcription factor Sox10, the neurotrophin receptor p75, Nestin and Slug [68], [69]. Recently, connexin 43 was suggested as a novel marker protein to selectively isolate remnant neural crest-derived stem cells from human adult periodontal ligament [70].

Purinergic receptors have been shown to be involved in neuronal development [71]. Suyama and coworkers [72] studied the role of extracellular purinergic signaling in cell-cell communications as well as physical cell-cell contacts during the proliferation and fate determination of neural stem cells (NSCs), which are persisting in the subventricular zone (SVZ) of adult mammalian brain. Based on their findings they suggested that purinergic signaling via the P2Y1 receptor promotes the proliferation of cells in the SVZ niche and thus is important for maintenance of adult neuronal differentiation in this niche. NSCs develop into neural progenitor cells (NPCs) which possess a limited self-renewal capacity and can differentiate towards neurons and glia [73]. ATP has been described as stimulating factor for NSC migration and NPC proliferation as well as negative regulator of terminal neuronal differentiation through P2 receptor signaling [74], [75]. Moreover, axonal elongation was shown to be modulated through a crosstalk between P2X7, P2Y1, and P2Y13 receptor subtypes [76].

#### 3.3.3 Mesenchymal stem cells

Mesenchymal stem cells (MSC) are multipotent cells that can be obtained from several tissues including bone marrow, umbilical cord, peripheral blood, wisdom teeth, and fat [5], [77], [78], [79], [80]. To define these cells most researchers agree and rely on the minimal criteria defined by the International Society of Cellular Therapy where MSCs should adhere to plastic under standard culture conditions, and must be able to differentiate into osteoblasts, adipocytes and chondroblasts under standard *in vitro* differentiating conditions, as confirmed by specific stainings. Additionally, a positive expression pattern (> 95% of the cells) of CD73, CD90, and CD105 is required as well as the absent expression (> 98% of the cells) of CD45, CD34, CD14 or CD11b, CD79 or CD19, and HLA-DR [81]. Purinergic signaling in mesenchymal stem cells aroused keenness interest because of the high availability of these cells due to their sources named above.

## 4 Purinergic receptors in mesenchymal stem cells

Mesenchymal stem cells play an important role in maintaining the homeostasis of mesodermal tissues throughout the adult body. Furthermore MSCs produce signaling molecules that are required for their active crosstalk in tissue environments. Among them are extracellular nucleotides and their metabolites which are more and more the focus of attention [82]. These molecules activate both, ionotropic and metabotropic receptors and thereby mediate fundamental cellular processes such as MSC proliferation, differentiation, and survival [32], [83]. In detail, Kawano and colleagues [15] reported that ATP autocrine/paracrine signaling induced calcium oscillations in undifferentiated human MSCs. Enzymatical hydrolysis of extracellular nucleotides by ecto-nucleoside triphosphate diphosphohydrolases (E-NTPDases) and ecto-5'-nucleotidase (CD73) generates a cellular signaling cascade essential for development and maintenance of MSCs [84]. Several research groups described an immunsuppressive effect of MSCs based on an increased adenosine production (mainly mediated by CD39, CD73, and adenosine deaminase) and signaling via adenosine A2A receptor [85], [86], [87]. Huicong and coworkers [88] found that targeted MSC transplantation corrected the imbalanced expression between adenosine A1 and A2A receptors in an epilepsy model.

**Fig. 3:**
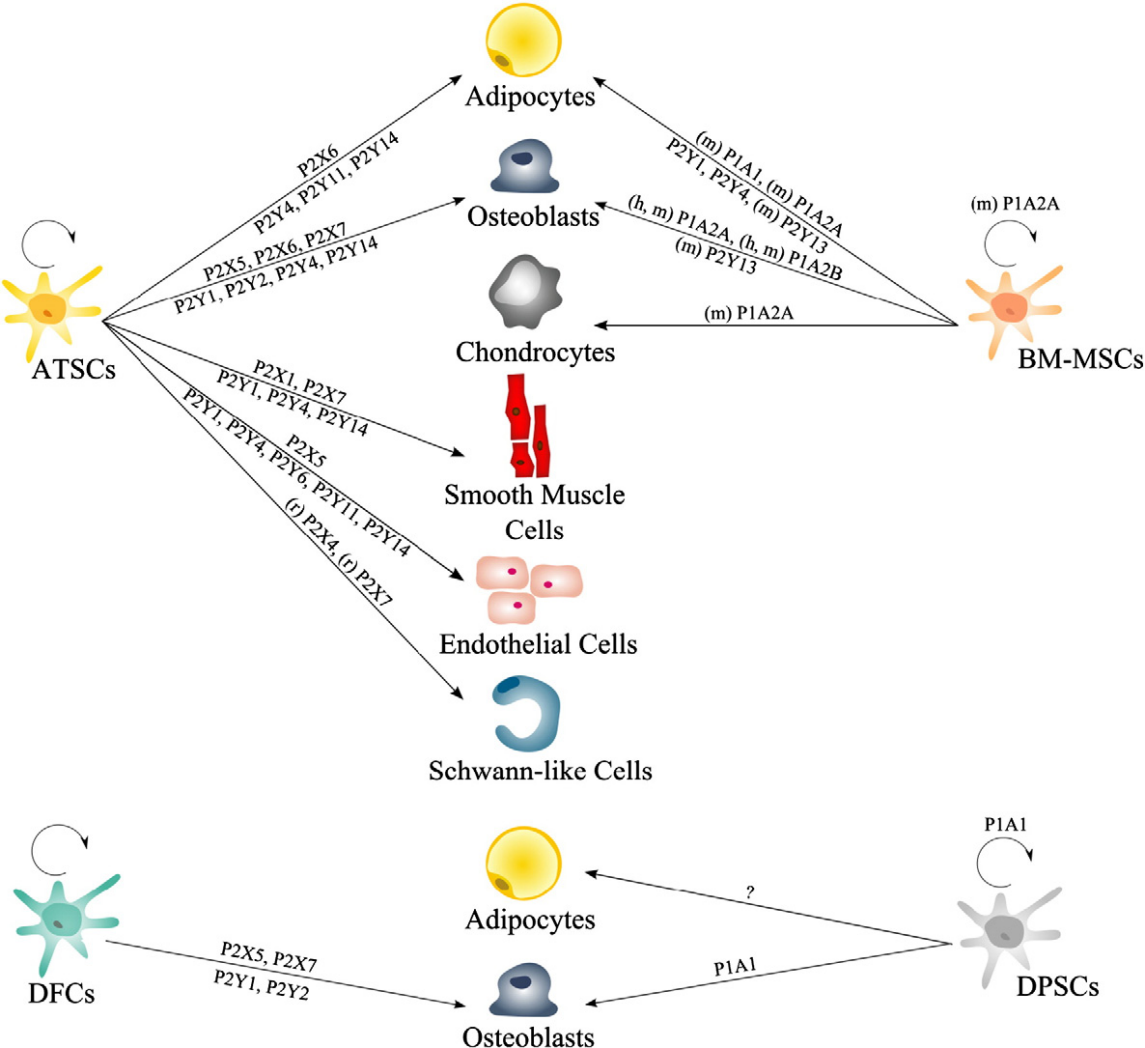
Distribution of purinergic receptors during MSC proliferation and differentiation. Summarized are data for purinergic receptors during proliferation and differentiation of MSCs originating from different sources. Dental pulp-derived stem cells (DPSCs), adipose tissue-derived stem cells (ATSC), bone marrow-derived mesenchymal stem cells (BM-MSC), dental follicle-derived cells (DFC), (m) mouse, (h) human, (r) rat, if not stated otherwise: human, on top P1 and P2X, underneath the arrow P2Y.

Only recently, it has been shown that MSC cell surface bound purinergic receptors and nucleotide processing ectoenzymes are also involved in the regulation of stem cell fate [55], [89]. MSC commitment towards a desired stem cell-derived tissue cell type might be induced by using selective purinergic receptor ligands. The better understanding of the mechanisms underlying MSC proliferation and differentiation might lead to an improved application of MSCs in regenerative medicine (Fig. 3) [5].

### 4.1 Purinergic receptors during MSC proliferation

Since ATP can be found in almost every living cell [90] and purinergic signaling was shown to be involved in stem cell development, there is a growing interest in this research area [32].

For example, several studies have indicated that ATP is spontaneously released from human MSCs (hMSCs) in culture [91]. Coppi and colleagues [16] demonstrated a decreasing proliferation rate in hMSCs upon ATP release which stimulated P2Y and P2X receptors. Similar, in another study the finding of a decreased proliferation rate of hMSCs after spontaneous ATP release in early stages of culture has been confirmed. There it was hypothesized that increased hMSC differentiation might be responsible for an ATP-induced decrease in proliferation [91]. Based on data from studies focusing on gene expression profiling it could also be shown that genes involved in cell proliferation of hMSCs were down-regulated upon ATP-stimulation, supporting the hypothesis that ATP decreases cell proliferation of hMSCs. Additionally, a strong up-regulation of genes involved in cell migration was found [92], which was confirmed in a more recent *in vivo* and *in vitro* study using bone marrow-derived mesenchymal stem cells (BM-MSC) [93]. By contrast, Riddle and coworkers [94] found that ATP increases cellular proliferation of bone marrow stromal cells, suggesting that extracellular ATP is required for fluid flow-induced increases in intracellular calcium concentration activating proliferation. The role of calcium was also investigated in an earlier study demonstrating that an ATP dependent autocrine/paracrine signaling pathway is involved in calcium ion oscillations that are known to play a pivotal role in differentiation and proliferation of hMSCs. ATP was found to stimulate P2Y1 receptors activating PLC-β to produce IP3 which induces calcium release [15]. In a more recent study it was demonstrated that NAD^+^ activates the P2Y11 receptor and a cAMP/cyclic ADP-ribose/[Ca^2 +^]_(i)_ signaling cascade which leads to the opening of L-type calcium channels. Furthermore it was shown that NAD^+^, either extracellularly added or autocrinally released, stimulates MSC functions, among them proliferation [95].

The role of P1 receptors in MSC proliferation is not well studied. There is some evidence suggesting that adenosine and the adenosine A2A receptor are important mediators in stimulating proliferation and differentiation of mouse BM-MSCs [96]. Whereas in another study the addition of an A1 receptor agonist (2-chloro-*N*(6)-cyclopentyl-adenosine, CCPA) to undifferentiated dental pulp-derived mesenchymal stem cells (DPSCs) showed no modification of proliferation in contrast to DPSCs induced towards the osteogenic lineage that revealed a significantly increased proliferation after eight days *in vitro* [97].

### 4.2 P1 receptors during MSC differentiation

Over the last decade, the presence and function of adenosine receptors on the plasma membrane of MSCs has been investigated in several studies. Adenosine, the natural ligand of the P1 receptors, can either be endogenously released or generated via enzymatic degradation of adenine nucleotides by E-NTPDases and ecto-5’-nucleotidase (CD73) [14], [84]. Upon stimulation, MSCs have been reported to actively secrete nucleotides such as ATP and NAD^+^ in order to modulate MSC functions e.g. proliferation, migration, and immunosuppression of activated T lymphocytes [16], [95].

Since some time there has been increased interest for the role of adenosine and its receptors in bone formation and remodeling [98]. It has been demonstrated that adenosine receptor signaling via cyclic AMP contributes to MSC differentiation towards chondrocytes and osteoblasts [99]. Cyclic-compressive loading of murine BM-MSCs reduced the expression of the ecto-5′-nucleotidase which acts as a regulatory factor in osteo-/chondrogenic differentiation via adenosine A2A receptor signaling [100]. Furthermore, it has been reported in several *in vitro* or *in vivo* studies that adenosine acts as an autocrine/paracrine signaling molecule that induces osteogenic differentiation of murine as well as human BM-MSCs via adenosine A2B receptor stimulation [93], [101], [102], [103]. Gharibi and colleagues [104] showed a predominant association of the osteoblast differentiation of murine BM-MSCs with adenosine A2B receptor expression and activation promoting the three stages of initiation, maturation and mineralization. The influence of the adenosine A1 receptor in osteogenesis is not quite clear until now. D’Alimonte and coworkers observed that stimulation of the adenosine A1 receptor enhanced the differentiation of human DPSCs towards osteoblasts via activation of the Wnt receptor signaling pathway [97]. In contrast to that, adenosine A1 receptor-knockout mice revealed no changes in osteoblast morphology and bone formation rates [105]. Moreover, it appeared that adenosine A1 receptor blockade or deletion could prevent ovariectomy-induced bone loss through diminishing osteoclast differentiation and function. This, in turn, is in line with a study performed by He and colleagues [102]. They found that osteoclast differentiation of human bone marrow-derived mononuclear cells from patients with osteolytic bone lesions was inhibited when treated with the adenosine A1 receptor antagonist rolofylline and A2B receptor agonist BAY60-6583.

Besides fat storage, adipose tissue is since some time considered to exert endocrine functions [106]. Adipocytes secrete adipokines such as adiponectin which has anti-inflammatory effects and seems to protect against atherosclerosis [107]. Exploring the underlying signaling pathways of adipogenesis is fundamental to a better understanding of adipose tissue development and remodeling [108]. Gharibi and colleagues investigated the expression of adenosine receptors during MSC adipogenesis and found that the adenosine A1 receptor is mainly involved in the lipogenic activity of adipocytes whereas the expression of the adenosine A2A receptor enhanced adipocytic differentiation and lipid accumulation [104]. Recently it was reported that the A2B receptor subtype mediates inhibition of adipogenesis through a novel signaling pathway involving Krüppel-like factor 4, a known regulator of stem cell maintenance [109], [110].

In addition to its prominent role in regulating MSC osteogenesis versus adipogenesis, adenosine has also been shown to induce the expression of hepatocyte-specific genes in mouse and human BM-MSCs *in vitro* [111]. Mohamadnejad and coworkers demonstrated that the inhibition of the hepatocyte growth factor-induced chemotaxis of BM-MSCs is mediated via adenosine A2A receptor signaling. Moreover, topical application of an exogenous adenosine A2A receptor agonist has been reported to promote wound healing via enhancement of local vessel sprouting and vasculogenesis in the early stages of tissue regeneration through recruitment of bone marrow-derived endothelial cells [112] (Table 1).

### 4.3 P2 receptors during MSC differentiation

Only quite recently, researchers working on adult stem cells have given more attention to endogenous release of nucleotides and the role of purinergic 2 receptors during MSC differentiation. A better understanding of the cellular and molecular mechanisms underlying MSC differentiation is crucial for safe application of adult stem cells in regenerative medicine.

In 2003 first data indicated that purinergic receptors are involved in stem cell lineage commitment towards osteoblasts. Ke and colleagues showed that P2X7 receptor knockout mice displayed a reduced periosteal bone formation rate and an increased trabecular bone resorption. [113]. Li and collaborators [114] demonstrated shortly after that the mechanically induced release of prostaglandins by MC3T3-E1 osteoblasts and MLO-Y4 osteocytes is also mediated via P2X7 receptor signaling. In another study it was published that preincubation of proliferating preadipose cell line 3 T3-L1 cells with extracellular ATP before addition of adipogenic induction medium resulted in an enhanced migration as well as an increased gene expression of adipose protein 2 compared to preadipocytes without ATP pretreatment [115]. Further, cell migration assays revealed that ATP induced actin filament reorganization and membrane ruffling, both mediated through P2Y receptor signaling, in a concentration dependent manner.

Recently, we investigated the role of several P2 receptors during differentiation of adipose tissue-derived MSCs (ATSCs) and ectomesenchymal dental follicle-derived cells (DFCs) [25]. In particular, we found the receptor subtypes P2X6, P2Y4 and P2Y14 to be key regulators in early lineage commitment, as they were regulated on gene and protein level at the branching point of adipogenic and osteogenic differentiation. Furthermore, P2X5, P2X7, P2Y1 and P2Y2 seem to be crucial for osteogenesis, whereas P2Y11 is involved in differentiation towards adipocytes. Since then, the number of data focusing on P2 receptor signaling in MSC differentiation is continuously rising. Ciciarello and collaborators [93] described that adipogenesis of BM-MSCs is mainly mediated through P2Y1 and P2Y4 receptor signaling. During stem cell differentiation towards adipocytes, ATP significantly increased the gene expression level of peroxisome proliferator-activated receptor-gamma (PPARγ2) and the accumulation of lipid droplets. Biver’s group reported that adipogenic induced bone marrow stromal cells derived from P2Y13 receptor-deficient mice (P2Y13R(−/−)) displayed an increased gene expression level of the adipogenic markers PPARγ2 and Adipsin and a higher number of adipocytes compared to P2Y13R(+/+)-derived MSCs [116]. In contrast, ADP stimulation of P2Y13R(−/−)-derived MSCs resulted in an decreased gene expression of the osteoblastic markers osterix, alkaline phosphatase, and type I collagen. Taken together, the receptor subtype P2Y13 seemed to be a notable key factor in the forking of osteoblast and adipocyte differentiation of bone marrow progenitors. In further studies a probable connection between MSC osteogenesis and P2Y13 receptor signaling was reaffirmed. Mechanical loading of knockout P2Y13R(−/−) mice tibiae resulted in increased bone formation and mineral accumulation rates compared to wild type control animals [117]. More recent data evidenced an age-dependent change of the skeletal phenotype in P2Y13 receptor-knockout mice compared to wild type control animals which was associated with altered serum fibroblast growth factor 23 and phosphorus levels [118].

Uracil nucleotides have been reported to regulate the osteogenic differentiation of primary bone marrow stromal cells from postmenopausal women, predominantly through stimulation of the P2Y6 receptor subtype which is linked to an increased intracellular Ca^2 +^ level [119]. Next to this certain polymorphic variants of the P2X7 receptor gene are associated with low lumbar spine bone mineral density and a greater risk of developing osteoporosis in post-menopausal women [120]. In addition the loss of P2X7 receptor function resulted in an altered adipocyte distribution and lipid accumulation in male but not in female mice *in vivo* [121]. Beaucage and coworkers revealed that the P2X7 receptor subtype might be involved in an age- and gender-dependent regulation of adipogenesis and lipid metabolism. Upon stimulation with extracellular ATP, adipocytes release several proinflammatory cytokines such as TNFα and IL-6 [122]. The characteristical inflammatory status of patients with metabolic syndrome has therefore been associated with an enhanced adipocyte P2X7 receptor expression in these subjects. Taken together P2X7 seems to be a major factor in adipogenic differentiation.

Endothelial and smooth muscle cells are the main cell types involved in cardiovascular physiology [123]. Damage or dysfunction of these cells may result in pathological processes such as atherosclerosis and hypertension, leading to cardiovascular diseases i.e. heart attack and stroke. MSCs have been extensively explored for their application in vascular tissue engineering [124], [125]. There is some evidence for the role of purinergic signaling in vascular cell proliferation and death [87], [126], but little is known with respect to the participation of purinergic receptors in MSC commitment towards endothelial and smooth muscle cells. Very recent data, achieved by using P2 receptor agonists and antagonists, gave new insights into the functional role of purinergic receptor regulation during endothelial and smooth muscle cell differentiation of adipose tissue-derived MSCs. We reported the up-regulation of the P2Y4 and P2Y14 receptor subtypes in both differentiation processes suggesting that these two receptors are important in early lineage commitment of MSCs towards vascular cell types [123].

The presence of Schwann cells is a critical limiting factor in nerve injury recovery. As an alternative source for peripheral nerve tissue engineering, MSCs were evaluated for their differentiation potential towards a Schwann cell phenotype [127]. Although there are several publications addressing purinergic signaling in primary Schwann cells, only few data exist with regard to the involvement of purinergic signaling during the developmental process of MSCs towards this phenotype [128]. During differentiation of adipose tissue-derived MSCs towards that specific cell lineage, the gene expression of P2X4 and P2X7 receptor was found to be upregulated [129]. ATP stimulation of these purinoreceptors triggers intracellular Ca^2 +^ signals and indicated towards the presence of a functional P2X7 receptor which is involved in control of cell death and survival. This is in line with the finding that P2X7 receptor signaling contributes to the death of Schwann cells transplanted into the spinal cord [130] (Table 2).

## 5 Summary and outlook

Over the last decade there was much progress in stem cell research. Especially, mesenchymal stem cells obtained from several adult tissues have been extensively studied with respect to their characterization, differentiation potential and immunomodulatory properties. In addition, signaling molecules that are involved in the determination of stem cell fate such as growth factors, Hox gene proteins, and chemokines have been well studied and already applied in clinical trials. Even though purinergic receptors are expressed in a wide range of cell types and huge amounts of data on the members of this family in various cellular functions have been published so far, there still is only sparse information on the role of purinergic receptor expression and regulation during stem cell proliferation and differentiation.

In this review, we outlined the latest results of the involvement of purinergic receptors in MSCs undergoing differentiation towards different distinct cell types e.g. adipocytes, osteoblasts, endothelial cells, smooth muscle cells or hepatocytes but more research is clearly needed. Unveiling the crosstalk of signaling pathways downstream of receptor activation will help to better understand how these ubiquitous expressed receptors exert their impact in (patho)physiological processes based on stem cell actions. More details on the cellular and molecular level still need to be attained to define new targets for drug development and to establish novel medical approaches with these cells. In the future, MSCs might be applied routinely to reconstruct or replace a variety of tissues and organs. Triggering them towards the desired lineage by using artificial purinergic receptor ligands might be an additional step to diminish the risk that a minor percentage of the stem cells stay undifferentiated and may develop towards a tumor. Regarding diseases or disorders originating from purinergic receptor dysfunction, the creation of iPS cells of patients suffering from such a disease might help to recapitulate the underlying cellular processes and clarify unclear issues. Later on, an improved stem cell lineage commitment combined with natural or synthetic scaffolds mimicking the tissues natural microenvironment will provide innovative stem cell-based regenerative approaches for future medicine. Last not least it should be noted that at the moment the benefit of MSCs in clinical trials seems not to be due to cell replacement but paracrine signaling which might involve secreted nucleotides.

## Competing interests

The authors have declared that no competing interests exist.

## Figures and Tables

**Table 1:** Involvement of P1 receptors during MSC differentiation.

P1 receptor	Present in	Involved in	References
A1	(h) DPSCs	Osteogenesis	[97]
A1	(h) BM-derived mononuclear cells	Osteogenesis	[102]
A2A	(m) BM-MSCs	Osteogenesis, chondrogenesis	[100]
A2A	(m) BM-MSCs	Adipogenesis	[104]
A2A	Mouse organism	Enhancement of local vessel sprouting	[112]
A2B	(m) BM-MSCs	Osteogenesis	[101], [104]
A2B	(h) BM-MSCs	Osteogenesis	[93], [102], [103]
A2B	(h) BM-derived mononuclear cells	Osteoclasts	[102]

Abbreviations: dental pulp-derived stem cells (DPSCs); bone marrow (BM); bone marrow-derived mesenchymal stem cells (BM-MSCs); human (h); mouse (m).

**Table 2:** Involvement of P2 receptors during MSC differentiation.

Purinergic receptor	Present in	Involved in	References
P2X4	(r) ATSCs	Schwann-like cell differentiation	[129]
P2X5	(h) ATSCs, DFCs	Osteogenesis	[25]
P2X6	(h) ATSCs	Osteogenesis, adipogenesis	[25]
P2X7	(h) ATSCs, DFCs	Osteogenesis	[25]
P2X7	(h) Adipocytes	Adipogenesis	[122]
P2X7	(m) Adipocytes	Adipogenesis	[121]
P2X7	(r) ATSCs	Schwann-like cell differentiation	[129]
P2X7	Mouse organism	Osteogenesis	[113], [114]
P2Y1	(h) ATSCs, DFCs	Osteogenesis	[25]
P2Y1	(h) BM-MSCs	Adipogenesis	[93]
P2Y2	(h) ATSCs, DFCs	Osteogenesis	[25]
P2Y4	(h) ATSCs	Osteogenesis, adipogenesis	[25]
P2Y4	(h) BM-MSCs	Adipogenesis	[93]
P2Y4	(h) ATSCs	Endothelial and smooth muscle cell differentiation	[123]
P2Y11	(h) ATSCs	Adipogenesis	[25]
P2Y13	(m) BM-MSCs	Osteogenesis, adipogenesis	[116]
P2Y13	(m) Osteoblasts	Osteogenesis	[117]
P2Y14	(h) ATSCs	Osteogenesis, adipogenesis	[25]
P2Y14	(h) ATSCs	Endothelial and smooth muscle cell differentiation	[123]

Abbreviations: Adipose tissue-derived stem cells (ATSCs); bone marrow-derived mesenchymal stem cells (BM-MSCs); dental follicle-derived cells (DFCs); human (h); mouse (m); rat (r).
